# Some new lacunary statistical convergence with ideals

**DOI:** 10.1186/s13660-016-1284-9

**Published:** 2017-01-10

**Authors:** Adem Kilicman, Stuti Borgohain

**Affiliations:** 1 Department of Mathematics, University Putra Malaysia, Serdang, 43400 Selangor, Malaysia; 2 Department of Mathematics, Indian Institute of Technology, Bombay, Powai, 400076 Mumbai, India

**Keywords:** Musielak-Orlicz function, ideal convergence, lacunary sequences, probabilistic normed space

## Abstract

In this paper, the idea of lacunary $I_{\lambda}$-statistical convergent sequence spaces is discussed which is defined by a Musielak-Orlicz function. We study relations between lacunary $I_{\lambda}$-statistical convergence with lacunary $I_{\lambda}$-summable sequences. Moreover, we study the $I_{\lambda}$-lacunary statistical convergence in probabilistic normed space and discuss some topological properties.

## Introduction

The concept of statistical convergence [1] which is the extended idea of convergence of real sequences has become an important tool in many branches of mathematics. For references one may see [2–8] and many more.

Similarly, *I*-convergence is also an extended notion of statistical convergence ([9]) of real sequences. A family of sets $I \subseteq2^{A}$ (power sets of *A*) is an ideal if *I* is additive, *i.e.* $S , T \in I \Rightarrow S \cup T \in I$, and hereditary *i.e.* $S \in I$, $T \subseteq S \Rightarrow T \in I$, where *A* is any non-empty set.

A lacunary sequence is an increasing integer sequence $\theta= (i_{j})$ such that $i_{0}=0$ and $h_{j}=i_{j}-i_{j-1} \rightarrow\infty$ as $j \rightarrow\infty$. As regards ideal convergence and lacunary ideal convergence, one may refer to [10–19] etc.

Note: Throughout this paper, *θ* will be determined by the interval $K_{j}=(k_{j-1}, k_{j}]$ and the ratio $\frac{k_{j}}{k_{j-1}}$ will be defined by $\phi_{j}$.

## Preliminary concepts

A sequence $(x_{i})$ of real numbers is statistically convergent to *M* if, for arbitrary $\xi>0$, the set $K(\xi)=\{i \in\mathbb{N}: \vert x_{i} -M \vert\geq\xi\}$ has natural density zero, *i.e.*,

$$\lim_{i} \frac{1}{i} \sum_{j=1}^{i} \chi_{K(\xi)}(j)=0, $$

where $\chi_{K(\xi)}$ denotes the characteristic function of $K(\xi)$.

A sequence $(x_{i})$ of elements of $\mathbb{R}$ is *I*-convergent to $M \in\mathbb{R}$ if, for each $\xi>0$,

$$\bigl\{ i \in\mathbb{N}: \vert x_{i} -M \vert \geq\xi\bigr\} \in I. $$

For any lacunary sequence $\theta= (i_{j})$, the space $N_{\theta}$ is defined as (Freedman *et al.* [5])

$$N_{\theta}= \biggl\{ (x_{i}): \lim_{j \rightarrow\infty} i_{j}^{-1} \sum_{i \in K_{j}} \vert x_{i} - M \vert =0, \mbox{ for some } M \biggr\} . $$

The concept of a Musielak-Orlicz function is defined as $\mathscr {M}=(M_{j})$. The sequence $\mathscr{N}=(N_{i})$ is defined by

$$N_{i}(a)=\sup\bigl\{ \vert a \vert b -M_{j}(b): b \geq0 \bigr\} ,\quad i=1,2,\ldots, $$

which is named the complementary function of a Musielak-Orlicz function $\mathscr{M}$ (see [20]) (throughout the paper $\mathscr{M}$ is a Musielak-Orlicz function).

If $\lambda=(\lambda_{i})$ is a non-decreasing sequence of positive integers such that Λ denotes the set of all non-decreasing sequences of positive integers. We call a sequence $\{x_{i} \}_{i \in \mathbb{N}}$ lacunary $I_{\lambda}$-statistically convergent of order *α* to *M*, if, for each $\gamma>0$ and $\xi>0$,

$$\biggl\{ i \in\mathbb{N}: \frac{1}{\lambda_{i}^{\alpha}}\biggl\vert \biggl\{ j \leq i: \frac{1}{h_{i}} \sum_{j \in I_{i}} M_{j} \biggl( \frac{\vert x_{j}-M \vert }{\rho^{(j)}} \biggr) \geq\gamma \biggr\} \biggr\vert \geq\xi \biggr\} \in I. $$

We denote the class of all lacunary $I_{\lambda}$-statistically convergent sequences of order *α* defined by a Musielak-Orlicz function by $S^{\alpha}_{I_{\lambda}}(\mathscr{M}, \theta)$.

Some particular cases:

1. If $M_{j}(x)=M(x)$, for all $j \in\mathbb{N}$, then $S_{I_{\lambda}}^{\alpha}(\mathscr{M}, \theta)$ is reduced to $S_{I_{\lambda}}^{\alpha}(M, \theta)$.
  Also, if $M_{j}(x)=x$, for all $j \in\mathbb{N}$, then $S_{I_{\lambda}}^{\alpha}(\mathscr{M}, \theta)$ will be changed as $S_{I_{\lambda}}^{\alpha}(\theta)$.
2. If $\lambda_{i}=i$, for all $i \in\mathbb{N}$, then $S_{I_{\lambda}}^{\alpha}(\mathscr{M}, \theta)$ will be reduced to $S_{I}^{\alpha}(\mathscr {M}, \theta)$.
3. If $\alpha=1$, then *α*-density of any set is reduced to the natural density of the set. So, the set $S_{I_{\lambda}}^{\alpha}(\mathscr {M}, \theta)$ reduces to $S_{I_{\lambda}}(\mathscr{M}, \theta)$ for $\alpha=1$.
4. If $\theta=(2^{r})$ and $\alpha=1$, then $(x_{j})$ is said to be $I_{\lambda}$-statistically convergent defined by a Musielak-Orlicz function, *i.e.* $(x_{j}) \in S_{I_{\lambda}}(\mathscr{M})$.
5. if $M_{j}(x)=x$, $\theta=(2^{r})$, $\lambda_{j}=j$, $\alpha=1$, then $I_{\lambda}$-lacunary statistically convergence of order *α* defined by Musielak-Orlicz function reduces to *I*-statistical convergence.

In this article, we define the concept of lacunary $I_{\lambda}$-statistically convergence of order *α* defined by $\mathscr{M}$ and investigate some results on these sequences. Later on, we investigate some results of lacunary $I_{\lambda}$-statistically convergence of real sequences in probabilistic normed space too.

## Main results

### Theorem 3.1

*Let* $\lambda=(\lambda_{i})$ *and* $\mu=(\mu_{i})$ *be two sequences in* Λ *such that* $\lambda_{i} \leq\mu_{i}$ *for all* $i \in \mathbb{N}$ *and* $0<\alpha\leq\beta\leq1$ *for fixed reals* *α* *and* *β*. *If* $\lim\inf_{i \rightarrow\infty} \frac{\lambda_{i}^{\alpha}}{\mu_{i}^{\beta}} >0$, *then* $S_{I_{\mu}}^{\beta}(\mathscr{M}, \theta) \subseteq S_{I_{\lambda}}^{\alpha}(\mathscr{M}, \theta)$.

### Proof

Suppose that $\lambda_{i} \leq\mu_{i}$ for all $i \in\mathbb {N}$ and $\lim\inf_{i \rightarrow\infty} \frac{\lambda_{i}^{\alpha}}{\mu _{i}^{\beta}} >0$. Since $I_{i} \subset J_{i}$, where $J_{i}=[i-\mu_{i}+1, i]$, so for $\gamma>0$, we can write

$$\bigl\{ j \in J_{i}: \vert x_{j} -M \vert \geq\gamma \bigr\} \supset\bigl\{ j \in I_{i}:\vert x_{j} -M \vert \geq\gamma\bigr\} , $$

which implies

$$\frac{1}{\mu_{i}^{\beta}} \bigl\vert \bigl\{ j \in J_{i}: \vert x_{j} - M \vert \geq\gamma \bigr\} \bigr\vert \geq\frac{\lambda_{i}^{\alpha}}{\mu _{i}^{\beta}}. \frac{1}{\lambda_{i}^{\alpha}} \bigl\vert \bigl\{ j \in I_{i}:\vert x_{j} - M \vert \geq\gamma \bigr\} \bigr\vert , $$

for all $i \in\mathbb{N}$.

Assume that $\lim\inf_{i \rightarrow\infty} \frac{\lambda_{i}^{\alpha}}{\mu_{i}^{\beta}}=a$, so from the definition we see that $\{i \in \mathbb{B}: \frac{\lambda_{i}^{\alpha}}{\mu_{i}^{\beta}} <\frac{a}{2} \}$ is finite. Now for $\xi>0$,

$$\begin{aligned} \biggl\{ i \in\mathbb{N}:\frac{1}{\lambda_{i}^{\beta}} \bigl\vert \bigl\{ j \in J_{i}: \vert x_{j} -M \vert \geq\gamma \bigr\} \bigr\vert \geq\xi \biggr\} \subset& \biggl\{ i \in\mathbb{N}: \frac{1}{\mu_{i}^{\alpha}} \bigl\vert \bigl\{ j \in I_{i} : \vert x_{j} -M \vert \geq\gamma \bigr\} \bigr\vert \geq\frac{a}{2} \xi \biggr\} \\ &{} \cup \biggl\{ i \in\mathbb{N} : \frac{\lambda_{i}^{\alpha}}{\mu_{i}^{\beta}} < \frac{a}{2} \biggr\} . \end{aligned}$$

Since *I* is admissible and $(x_{j})$ is a lacunary $I_{\mu}$-statistically convergent sequence of order *β* defined by $\mathscr{M}$, by using the continuity of $\mathscr{M}$, we see with the lacunary sequence $\theta=(h_{i})$, the right hand side belongs to *I*, which completes the proof. □

### Theorem 3.2

*If* $\lim_{i \rightarrow\infty} \frac{\mu_{i}}{\lambda _{i}^{\beta}}=1$, *for* $\lambda=(\lambda_{i})$ *and* $\mu=(\mu_{i})$ *two sequences of* Λ *such that* $\lambda_{i} \leq\mu_{i}$, $\forall i \in\mathbb{N}$ *and* $0<\alpha\leq\beta\leq1$ *for fixed* *α*, *β* *reals*, *then* $S_{I_{\lambda}}^{\alpha}(\mathscr{M}, \theta) \subseteq S_{I_{\mu}}^{\beta}(\mathscr{M}, \theta)$.

### Proof

Let $(x_{j})$ be lacunary $I_{\lambda}$-statistically convergent to *M* of order *α* defined by $\mathscr{M}$. Also assume that $\lim_{i \rightarrow\infty} \frac{\mu_{i}}{\lambda_{i}^{\beta}} =1$. Choose $m \in\mathbb{N}$ such that $\vert \frac{\mu _{i}}{\lambda_{i}^{\beta}}-1 \vert < \frac{\xi}{2}$, $\forall i\geq m$.

Since $I_{i} \subset J_{i}$, for $\gamma>0$, we may write

$$\begin{aligned} \frac{1}{\mu_{i}^{\beta}} \bigl\vert \bigl\{ j \in J_{i}: \vert x_{j} -M \vert \geq\gamma \bigr\} \bigr\vert =& \frac{1}{\mu_{i}^{\beta}} \bigl\vert \bigl\{ i- \mu_{i}+1 \leq j \leq i-\lambda_{i} : \vert x_{j} -M \vert \geq\gamma \bigr\} \bigr\vert \\ &{}+ \frac{1}{\mu_{i}^{\beta}} \bigl\vert \bigl\{ j \in I_{i} :\vert x_{j} -M\vert\geq\gamma \bigr\} \bigr\vert \\ \leq& \frac{\mu_{i}-\lambda_{i}}{\mu_{i}^{\beta}} + \frac{1}{\mu_{i}^{\beta}} \bigl\vert \bigl\{ j \in I_{i} : \vert x_{j} -M \vert\geq\gamma \bigr\} \bigr\vert \\ \leq& \frac{\mu_{i}-\lambda_{i}^{\beta}}{\lambda_{i}^{\beta}} + \frac{1}{\mu _{i}^{\beta}} \bigl\vert \bigl\{ j \in I_{i} : \vert x_{j} -M \vert\geq \gamma \bigr\} \bigr\vert \\ \leq& \biggl( \frac{\mu_{i}}{\lambda_{i}^{\beta}}-1 \biggr) + \frac{1}{\lambda _{i}^{\alpha}} \bigl\vert \bigl\{ j \in I_{i}: \vert x_{j} -M \vert\geq\gamma \bigr\} \bigr\vert \\ =& \frac{\xi}{2} + \frac{1}{\lambda_{i}^{\alpha}} \bigl\vert \bigl\{ j \in I_{i} : \vert x_{j} - M \vert \geq\gamma \bigr\} \bigr\vert . \end{aligned}$$

Hence,

$$\begin{aligned} \biggl\{ i \in\mathbb{N}:\frac{1}{\mu_{i}^{\beta}} \bigl\vert \bigl\{ j \leq i: \vert x_{j} -M \vert \geq\gamma \bigr\} \bigr\vert \geq\xi \biggr\} \subset& \biggl\{ i \in\mathbb{N}: \frac{1}{\lambda_{i}^{\alpha}} \bigl\vert \bigl\{ j \in I_{i} : \vert x_{j} -M \vert\geq\gamma \bigr\} \bigr\vert \geq \frac{\xi}{2} \biggr\} \\ &{}\cup \{ 1,2,3,\ldots,m \}. \end{aligned}$$

Since $(x_{j})$ is lacunary $I_{\lambda}$-statistically convergent sequence of order *α* defined by $\mathscr{M}$ and since *I* is admissible, by using the continuity of $\mathscr{M}$, it follows that the set on the right hand side with the lacunary sequence $\theta=(h_{i})$ belongs to *I* and

$$S_{I_{\lambda}}^{\alpha}(\mathscr{M}, \theta)\subseteq S_{I_{\mu}}^{\beta}(\mathscr{M}, \theta). $$

 □

We define the lacunary $I_{\lambda}$-summable sequence of order *α* defined by $\mathscr{M}$ as

$$w_{I_{\lambda}}^{\alpha}(\mathscr{M},\theta)= \biggl\{ i \in\mathbb{N}: \frac{1}{\lambda_{i}^{\alpha}} \biggl( j \leq i: \frac{1}{h_{i}} \sum _{j \in I_{i}} M_{j} \biggl( \frac{ \vert x_{j} - M \vert }{ \rho^{(j)}} \biggr) \geq \gamma \biggr) \biggr\} \in I. $$

### Theorem 3.3

*Given* $\lambda=(\lambda_{i})$, $\mu=(\mu_{i}) \in\Lambda$. *Suppose that* $\lambda_{i} \leq\mu_{i}$ *for all* $i \in\mathbb{N}$, $0 < \alpha\leq\beta\leq1$. *Then*:

1. *If* $\lim\inf_{i \rightarrow\infty} \frac{\lambda_{i}^{\alpha}}{\mu _{i}^{\beta}} >0$, *then* $w_{\mu}^{\beta}(\mathscr{M}, \theta) \subset w_{\lambda}^{\alpha}(\mathscr{M}, \theta)$.
2. *If* $\lim_{i \rightarrow\infty} \frac{\mu_{i}}{\lambda_{i}^{\beta}} = 1$, *then* $\ell_{\infty}\cap w_{\lambda}^{\alpha}(\mathscr{M},\theta) \subset w_{\mu}^{\beta}(\mathscr{M},\theta)$.

### Theorem 3.4

*Let* $\lambda_{i} \leq\mu_{i}$ *for all* $i \in\mathbb{N}$, *where* $\lambda, \mu\in\Lambda$. *Then*, *if* $\lim\inf_{i \rightarrow \infty} \frac{\lambda_{i}^{\alpha}}{\mu_{i}^{\beta}} >0$, *and if* $(x_{j})$ *is lacunary* $I_{\mu}$-*summable of order* *β* *defined by* $\mathscr{M}$, *then it is lacunary* $I_{\lambda}$-*statistically convergent of order* *α* *defined by* $\mathscr{M}$. *Here* $0 <\alpha\leq\beta\leq1$, *for fixed reals* *α* *and* *β*.

### Proof

For any $\gamma>0$, we have

$$\begin{aligned} \sum_{j \in J_{i}} \vert x_{j} -M \vert =& \sum_{j \in J_{i}, \vert x_{j} -M \vert\geq\varepsilon} \vert x_{j} -M \vert+ \sum _{j \in J_{i}, \vert x_{j} -M \vert< \varepsilon} \vert x_{j} -M \vert \\ \geq& \sum_{j \in I_{i}, \vert x_{j} - M \vert\geq\varepsilon} \vert x_{j} - M \vert+ \sum_{j \in I_{i}, \vert x_{j} -M \vert\geq\varepsilon} \vert x_{j} -M \vert \\ \geq& \sum_{j \in I_{i}, \vert x_{j} -M \vert\geq\varepsilon} \vert x_{j} -M \vert \\ \geq& \bigl\vert \bigl\{ j \in I_{i}: \vert x_{j} -M \vert\geq\gamma \bigr\} \bigr\vert . \gamma. \end{aligned}$$

Therefore,

$$\begin{aligned} \frac{1}{\mu_{i}^{\beta}} \sum_{j \in J_{i}} \vert x_{j} -M \vert \geq& \frac {1}{\mu_{i}^{\beta}} \bigl\vert \bigl\{ j \in I_{i} : \vert x_{j} - M \vert\geq\gamma \bigr\} \bigr\vert . \gamma \\ \geq&\frac{\lambda_{i}^{\alpha}}{\mu_{i}^{\beta}}. \frac{1}{\lambda_{i}^{\alpha}} \bigl\vert \bigl\{ j \in I_{i}: \vert x_{j} - M \vert\geq\gamma\bigr\} \bigr\vert . \gamma. \end{aligned}$$

If $\lim\inf_{i \rightarrow\infty} \frac{\lambda_{i}^{\alpha}}{\mu_{i}^{\beta}} =a$, then $\{ i \in\mathbb{N}: \frac{\lambda_{i}^{\alpha}}{\mu _{i}^{\beta}} < \frac{a}{2} \}$ is finite. So, for $\delta>0$, we get

$$\begin{aligned}& \biggl\{ i \in\mathbb{N}: \frac{1}{\lambda_{i}^{\alpha}}\biggl\vert \biggl\{ j \leq i: \sum _{j \in J_{i}} \vert x_{j} - M \vert\geq\gamma \biggr\} \biggr\vert \geq\xi \biggr\} \\& \quad \subset \biggl\{ i \in\mathbb{N}: \frac {1}{\mu_{i}^{\beta}} \bigl\{ j \in I_{i} : \vert x_{j} - M \vert \geq\gamma \bigr\} \geq\frac{a}{2} \xi \biggr\} \\& \qquad {}\cup \biggl\{ i \in\mathbb{N} : \frac{\lambda_{i}^{\alpha}}{\mu_{i}^{\beta}} < \frac{a}{2} \biggr\} . \end{aligned}$$

Since *I* is admissible and $(x_{j})$ is lacunary $I_{\mu}$-summable sequence of order *β* defined by $\mathscr{M}$, using its continuity and using the lacunary sequence $\theta=(h_{i})$, we can conclude that $w_{I_{\mu}}^{\beta}(\mathscr{M}, \theta) \subseteq S_{I_{\lambda}}^{\alpha}(\mathscr{M}, \theta)$. □

### Theorem 3.5

*Let* $\lim_{i \rightarrow\infty} \frac{\mu_{i}}{\lambda _{i}^{\beta}}=1$, *where* $0< \alpha\leq\beta\leq1$ *for fixed reals* *α* *and* *β* *and* $\lambda_{i} \leq\mu_{i}$, *for all* $i \in\mathbb {N}$, *where* $\lambda, \mu\in\Lambda$. *Also let* *θ*! *be a refinement of* *θ*. *Let* $(x_{j})$ *to be a bounded sequence*. *If* $(x_{j})$ *is lacunary* $I_{\lambda}$-*statistically convergent sequence of order* *α* *defined by* $\mathscr{M}$, *then it is also a lacunary* $I_{\mu}$-*summable sequence of order* *β* *defined by* $\mathscr{M}$. *i*.*e*. $S_{I_{\lambda}}^{\alpha}(\mathscr{M},\theta) \subseteq w_{I_{\mu}}^{\beta}(\mathscr{M}, \theta!)$.

### Proof

Suppose that $(x_{j})$ is lacunary $I_{\lambda}$-statistically convergent sequence of order *α* defined by $\mathscr{M}$.

Given that $\lim_{i \rightarrow\infty} \frac{\mu_{i}}{\lambda_{i}^{\beta}}=1$, we can choose $s \in\mathbb{N}$ such that $\vert \frac{\mu _{i}}{\lambda_{i}^{\beta}}-1 \vert < \frac{\delta}{2}$, $\forall i \geq s$.

Assume that there are a finite number of points $\theta!=(j_{i}^{!})$ in the interval $I_{i}=(j_{i-1}, j_{i}]$. Let there exists exactly one point $j_{i}^{!}$ of *θ*! in the interval $I_{i}$, that is, $j_{i-1}=j _{p-1}^{!} < j_{p}^{!} < j_{p+1}^{!}=j_{i}$, for $p \in\mathbb{N}$.

Let $I_{i}^{1}=(j_{i-1},j_{p}]$, $I_{i}^{2}=(j_{p}, j_{i}]$, $h_{i}^{1}=j_{p}-j_{i-1}$, $h_{i}^{2}=j_{i}-j_{p}$. Since $I_{i}^{1} \subset I_{i}$ and $I_{i}^{2} \subset I_{i}$, both $h_{i}^{1}$ and $h_{i}^{2}$ tend to ∞ as $i \rightarrow\infty$. We have

$$\begin{aligned}& \frac{1}{\mu_{i}^{\beta}} \biggl(h_{i}^{-1} \sum _{j \in J_{i}} \vert x_{j} -M \vert \biggr) \\& \quad \leq \frac{1}{\mu_{i}^{\beta}} \biggl(\bigl(h_{i}^{-1}h_{i}^{1} \bigr) \bigl(h_{i}^{1}\bigr)^{-1} \sum _{j \in I_{i}^{1}} \vert x_{j} -M \vert + \bigl(h_{i}^{-1}h_{i}^{2}\bigr) \bigl(h_{i}^{2}\bigr)^{-1} \sum _{j \in I_{i}^{2}} \vert x_{j} -M \vert \biggr) \\& \quad \leq \biggl(\frac{\mu_{i} - \lambda_{i}}{\mu_{i}^{\beta}} \biggr) \bigl(h_{i}^{-1}h_{i}^{1} \bigr) \bigl(h_{i}^{1}\bigr)^{-1}L+ \frac{1}{\mu_{i}^{\beta}} \biggl(\bigl(h_{i}^{-1}h_{i}^{2} \bigr) \bigl(h_{i}^{2}\bigr)^{-1} \sum _{j \in I_{i}^{2}} \vert x_{j} -M \vert \biggr) \\& \quad \leq \biggl(\frac{\mu_{i} - \lambda_{i}^{\beta}}{\lambda_{i}^{\beta}} \biggr) \bigl(h_{i}^{-1}h_{i}^{1} \bigr) \bigl(h_{i}^{1}\bigr)^{-1} L+ \frac{1}{\mu_{i}^{\beta}} \biggl(\bigl(h_{i}^{-1}h_{i}^{2} \bigr) \bigl(h_{i}^{2}\bigr)^{-1} \sum _{j \in I_{i}^{2}} \vert x_{j} -M \vert \biggr) \\& \quad \leq \biggl(\frac{\mu_{i} }{\lambda_{i}^{\beta}}-1 \biggr) \bigl(h_{i}^{-1}h_{i}^{1} \bigr) \bigl(h_{i}^{1}\bigr)^{-1} L+ \frac{1}{\mu_{i}^{\beta}} \biggl(\bigl(h_{i}^{-1}h_{i}^{2} \bigr) \bigl(h_{i}^{2}\bigr)^{-1} \sum _{j \in I_{i}^{2}, \vert x_{j} - M \vert \geq\varepsilon} \vert x_{j} -M \vert \biggr) \\& \qquad {} + \frac{1}{\mu_{i}^{\beta}} \biggl(\bigl(h_{i}^{-1}h_{i}^{2} \bigr) \bigl(h_{i}^{2}\bigr)^{-1} \sum _{j \in I_{i}^{2}, \vert x_{j} - M \vert < \varepsilon} \vert x_{j} -M \vert \biggr) \\& \quad \leq \biggl(\frac{\mu_{i}}{\lambda_{i}^{\beta}}-1 \biggr) \bigl(h_{i}^{-1}h_{i}^{1} \bigr) \bigl(h_{i}^{1}\bigr)^{-1} L+ \frac{L}{\lambda_{i}^{\alpha}} \bigl\vert \bigl\{ j \in I_{i}: \bigl(h_{i}^{-1}h_{i}^{2}\bigr) \bigl(h_{i}^{2}\bigr)^{-1} \vert x_{j}-M \vert \geq\varepsilon \bigr\} \bigr\vert \\& \qquad {} + \varepsilon\bigl(h_{i}^{-1}h_{i}^{2} \bigr) \bigl(h_{i}^{2}\bigr)^{-1},\quad \forall i \in \mathbb{N} \\& \quad = \frac{\delta}{2}\bigl(h_{i}^{-1}h_{i}^{1} \bigr) \bigl(h_{i}^{1}\bigr)^{-1} L+ \frac{L}{\lambda _{i}^{\alpha}} \bigl\vert \bigl\{ j \in I_{i}: \bigl(h_{i}^{-1}h_{i}^{2}\bigr) \bigl(h_{i}^{2}\bigr)^{-1} \vert x_{j}-M \vert \geq\varepsilon \bigr\} \bigr\vert + \varepsilon \bigl(h_{i}^{-1}h_{i}^{2} \bigr) \bigl(h_{i}^{2}\bigr)^{-1}. \end{aligned}$$

Since $x \in w_{I_{\mu}}^{\beta}(\mathscr{M}, \theta!)$, we have $0< h_{i}^{-1}h_{i}^{1}\leq1$ and $0< h_{i}^{-1}h_{i}^{2} \leq1$.

Hence, for $\xi>0$,

$$\begin{aligned} \biggl\{ i \in\mathbb{N}: \frac{1}{\mu_{i}^{\beta}} \biggl(\frac{1}{h_{i}} \sum _{j \in J_{i}} \vert x_{j}-M \vert \geq\gamma \biggr) \geq\xi \biggr\} &\subset \biggl\{ i \in\mathbb{N}: \frac{L}{\lambda_{i}^{\alpha}} \biggl\vert \biggl\{ j \in I_{i}: \frac{1}{h_{i}^{2}} \vert x_{j}-M \vert\geq\gamma \biggr\} \biggr\vert \geq\xi \biggr\} \\ &\quad {}\cup \{1,2,3,\ldots,s\}. \end{aligned} $$

Since $(x_{j})$ is lacunary $I_{\lambda}$-statistically convergent sequence of order *α* defined by $\mathscr{M}$ and since *I* is admissible, by using the continuity of $\mathscr{M}$, we can say that

$$S_{I_{\lambda}}^{\alpha}(\mathscr{M},\theta) \subseteq w_{I_{\mu}}^{\beta}(\mathscr{M}, \theta!). $$

 □

### Corollary 3.1

*Let* $\lambda \leq\mu_{i}$ *for all* $i \in\mathbb{N}$ *and* $0< \alpha\leq\beta\leq1$. *Let* $\lim\inf_{i \rightarrow\infty } \frac{\lambda_{i}^{\alpha}}{\mu_{i}^{\beta}} >0$, *θ*! *be the refinement of* *θ*. *Also let* $\mathscr{M}=(M_{i})$ *be a Musielak*-*Orlicz function where* $(M_{i})$ *is pointwise convergent*. *Then* $w_{I_{\mu}}^{\beta}(\mathscr {M}, \theta!)\subset S_{I_{\lambda}}^{\alpha}(\mathscr{M}, \theta)$ *iff* $\lim_{i} M_{i} (\frac{\gamma}{\rho^{(i)}} )>0$, *for some* $\gamma >0$, $\rho^{(i)}>0$.

### Corollary 3.2

*Let* $\mathscr{M}=(M_{i})$ *be a Musielak*-*Orlicz function and* $\lim_{i \rightarrow\infty} \frac{\mu_{i}}{\lambda_{i}^{\beta}} =1$, *for fixed numbers* *α* *and* *β* *such that* $0< \alpha\leq\beta \leq1$. *Then* $S_{I_{\lambda}}^{\alpha}(\mathscr{M}, \theta) \subset w_{I_{\mu}}^{\beta}(\mathscr{M}, \theta)$ *iff* $\sup_{\nu}\sup_{i} (\frac {\nu}{\rho^{(i)}} )$.

## Lacunary $I_{\lambda}$-statistical convergence in probabilistic normed spaces

Let *X* be a real linear space and $\nu: X \rightarrow D$, where *D* is the set of all distribution functions $g:\mathbb{R} \rightarrow\mathbb {R}_{0}^{+}$ such that it is non-decreasing and left-continuous with $\inf_{t \in\mathbb{R}} g(t)=0$ and $\sup_{t \in\mathbb{R}} g(t)=1$. The probabilistic norm or *ν*-norm is a *t*-norm [21] satisfying the following conditions:

1. $\nu_{p}(0)=0$,
2. $\nu_{p}(t)=1$ for all $t>0$ iff $p=0$,
3. $\nu_{\alpha p}(t)=\nu_{p} (\frac{t}{\vert \alpha \vert } )$ for all $\alpha\in\mathbb{R}\backslash\{0 \}$ and for all $t >0$,
4. $\nu_{p+q}(s+t) \geq \tau(\nu_{p}(s),\nu_{q}(t))$ for all $p,q \in X$ and $s,t \in\mathbb{R}_{0}^{+}$;

$(X,\nu, \tau)$ is named a probabilistic normed space, in short PNS.

A sequence $x=(x_{i})$ is *I*-convergent to $M \in X$ in $(X,\nu,\tau )$ for each $\xi>0$ and $t>0$, $\{ i \in\mathbb{N}: \nu_{x_{i}- M }(t) \leq1-\xi\} \in I$ (here *I* is a non-trivial ideal of $\mathbb {N}$) [19].

We define a sequence $x=(x_{i})$ to be lacunary $I_{\lambda}$-statistical convergent to *M* in $(X,\nu,\tau)$ defined by $\mathscr{M}$, if, for each $\nu>0$, $M>0$, $\mu>0$, $\xi>0$ and $t>0$,

$$\biggl\{ i \in\mathbb{N}: \frac{1}{\lambda_{i}} \biggl\vert \biggl\{ j \leq i: \frac{1}{h_{i}} \sum_{j \in I_{i}} M_{j} \biggl( \frac{\nu_{x_{j}-M}(t)}{\rho ^{(j)}} \biggr) \leq1-\mu \biggr\} \biggr\vert \leq1-\xi \biggr\} \in I. $$

We write it as $I_{\lambda}^{\nu}(\theta) \lim x=\psi$.

Example: Let $(\mathbb{R}, \nu, \tau)$ be a PNS with the probabilistic norm $\nu_{p}(t)=\frac{t}{t+\vert p\vert }$ (for all $p \in\mathbb{R}$ and every $t>0$) and $\tau(a,b) =ab$. Also, let *I* be a non-trivial admissible ideal such that $I=\{ B \subset\mathbb{N}: \delta(B)=0 \}$. Define a sequence *x* as follows:

$$x_{i} = \textstyle\begin{cases} \frac{1}{i} & \mbox{if $i=k^{2}$, $i \in\mathbb{N}$};\\ 0 & \mbox{otherwise}. \end{cases} $$

Then we have, for each $\nu>0$, $M>0$, $\mu>0$, $\xi>0$ and $t>0$, $\delta (K)=0$, where

$$K= \biggl\{ i \in\mathbb{N}: \frac{1}{\lambda_{i}} \biggl\vert \biggl\{ j \leq i: \frac{1}{h_{i}} \sum_{j \in I_{i}} M_{j} \biggl( \frac{\nu_{x_{j}-M }(t)}{\rho^{(j)}} \biggr) \leq1-\mu \biggr\} \biggr\vert \leq1-\xi \biggr\} , $$

which implies $K \in I$ and $I_{\lambda}^{\nu}(\theta)- \lim=0$.

### Theorem 4.1

*Let* $(X, \nu,\tau)$ *be a PNS*. *If* $x=(x_{i})$ *is lacunary* $I_{\lambda}^{\nu}$-*statistical convergent*, *then it has a unique limit*.

### Proof

Suppose $x=(x_{i})$ to be lacunary $I_{\lambda}^{\nu}$-statistical convergent in *X* which has two limits, $M_{1}$ and $M_{2}$.

For $\beta>0$ and $t>0$, let us choose $\xi>0$ such that $\tau((1-\xi), (1-\xi)) \geq1-\beta$.

Take the following sets:

$$\begin{aligned}& K_{1}(\xi,t)= \biggl\{ i \in\mathbb{N}: \frac{1}{\lambda_{i}}\biggl\vert \biggl\{ j \leq i: \frac{1}{h_{i}} \sum_{j \in I_{i}} M_{j} \biggl(\frac{\nu _{x_{j}-M_{1}}(t)}{\rho^{(j)}} \biggr) \leq1-\mu \biggr\} \biggr\vert \leq1-\xi \biggr\} , \\& K_{2}(\xi,t)= \biggl\{ i \in\mathbb{N}: \frac{1}{\lambda_{i}}\biggl\vert \biggl\{ j \leq i: \frac{1}{h_{i}} \sum_{j \in I_{i}} M_{j} \biggl(\frac{\nu _{x_{j}-M_{2}}(t)}{\rho^{(j)}} \biggr) \leq1-\mu \biggr\} \biggr\vert \leq1-\xi \biggr\} . \end{aligned}$$

Since $x=(x_{i})$ is lacunary $I_{\lambda}^{\nu}$-statistical convergent to $M_{1}$, we have $K_{1}(\xi,t) \in I$. Similarly, $K_{2}(\xi,t) \in I$.

Now, let $K(\xi,t)=K_{1}(\xi,t) \cup K_{2}(\xi,t) \in I$. We see that $K(\xi ,t)$ belongs to *I*, from which it is clear that $K^{C}(\xi,t)$ is non-empty set in $F(I)$, where $F(I)$ is the filter associated with the ideal *I* [9].

If $i \in K^{C}(\xi,t)$, then we have $i \in K_{1}^{C}(\xi,t) \cap K_{2}^{C}(\xi ,t)$ and so

$$\nu_{M_{1}-M_{2}}(t) \geq \tau \left(\nu_{x_{i}-M_{1}} \left( \frac {t}{2} \right) , \nu_{x_{i}-M_{2}} \left(\frac{t}{2} \right) \right) > \tau((1-\xi ) , (1-\xi)). $$

Since $\tau((1-\xi), (1-\xi)) \geq1-\beta$, it follows that $\nu_{M _{1}-M_{2}} (t) > 1-\beta$.

For arbitrary $\beta>0$, we get $\nu_{M_{1}-M_{2}} (t)=1$ for all $t>0$, which proves $M_{1}=M_{2}$. □

### Theorem 4.2

*Let* $(X, \nu, \tau)$ *be a PNS*. *If* *x* *is lacunary* $I^{\nu}$-*statistical convergent*, *then it is lacunary* $I_{\lambda}^{\nu}$-*statistical convergent if* $\lim_{i} \frac{\lambda_{i}}{i}>0$.

### Proof

For given $\mu>0$, $\xi>0$, and $t>0$,

$$\biggl\{ j \leq i: \frac{1}{h_{i}} \sum_{j \in I_{i}} M_{j} \biggl(\frac{\nu _{x_{j}-M}(t)}{\rho^{(j)}} \biggr) \leq1- \mu \biggr\} \supset \biggl\{ j \in I_{i} : \frac{1}{h_{i}} \sum _{j \in I_{i}} M_{j} \biggl(\frac{\nu_{x_{j}-M }(t)}{\rho^{(j)}} \biggr) \leq1- \mu \biggr\} . $$

Therefore,

$$\begin{aligned}& \frac{1}{i} \biggl\{ j \leq i: \frac{1}{h_{i}} \sum _{j \in I_{i}} M_{j} \biggl(\frac{\nu_{x_{j}-M}(t)}{\rho^{(j)}} \biggr) \leq1-\mu \biggr\} \\& \quad \geq \frac{1}{i} \biggl\{ j \in I_{i}: \frac{1}{h_{i}} \sum_{j \in I_{i}} M_{j} \biggl( \frac{\nu_{x_{j}-M}(t)}{\rho^{(j)}} \biggr) \leq1-\mu \biggr\} \\& \quad \geq \frac{1}{\lambda_{i}}.\frac{\lambda_{i}}{i} \biggl\{ j \in I_{i}: \frac {1}{h_{i}} \sum_{j \in I_{i}} M_{j} \biggl( \frac{\nu_{x_{j}-M}(t)}{\rho ^{(j)}} \biggr) \leq1-\mu \biggr\} , \\& \biggl\{ i \in\mathbb{N}: \frac{1}{i} \biggl\{ j \leq i: \frac{1}{h_{i}} \sum_{j \in I_{i}} M_{j} \biggl(\frac{\nu_{x_{j}-M}(t)}{\rho^{(j)}} \biggr) \leq1-\mu \biggr\} \leq1-\xi \biggr\} \\& \quad \geq\frac{\lambda_{i}}{i} \biggl\{ i \in\mathbb{N}: \frac{1}{\lambda_{i}} \biggl\{ j \in I_{i}: \frac{1}{h_{i}} \sum_{j \in I_{i}} M_{j} \biggl(\frac{\nu _{x_{j}-M}(t)}{\rho^{(j)}} \biggr) \leq1-\mu \biggr\} \leq1-\xi \biggr\} . \end{aligned}$$

Since $\lim_{i} \frac{\lambda_{i}}{i}>0$ and taking the limit $i \rightarrow\infty$, we get $I_{\lambda}^{\nu}(\theta)- \lim x=M$. □

We define $x=(x_{i})$ to be lacunary *λ*-statistically convergent to *M* with respect to *ν* as

$$\delta \biggl( \biggl\{ i \in\mathbb{N}: \frac{1}{\lambda_{i}} \biggl\vert \biggl\{ j \leq i: \frac{1}{h_{i}} \sum_{j \in I_{i}} M_{j} \biggl(\frac{\nu _{x_{j}-M}(t)}{\rho^{(j)}} \biggr) \leq1-\mu \biggr\} \biggr\vert \leq1-r \biggr\} \biggr)=0. $$

### Theorem 4.3

*Let* $(X,\nu,\tau)$ *be a PNS*.

1. *If* *x* *is lacunary* *λ*-*statistically convergent to* *M*, *then it is also lacunary* $I_{\lambda}^{\nu}$-*statistically convergent to* *M*.
2. *If* $I_{\lambda}^{\nu}(\theta)- \lim x=M_{1}$, $I_{\lambda}^{\nu}(\theta)- \lim y=M_{2}$, *then* $I_{\lambda}^{\nu}(\theta)- \lim(x_{k}+y_{k})=(M_{1}+M_{2})$.
3. *If* $I_{\lambda}^{\nu}(\theta)- \lim x=M$,*then* $I_{\lambda}^{\nu}(\theta )- \lim\alpha x=\alpha M$.

### Theorem 4.4

*Let* $(X,\nu, \tau)$ *be a PNS*. *If* *x* *is lacunary* *λ*-*statistical convergent to* *M*, *then* $I_{\lambda}^{\nu}(\theta )- \lim x=M$.

### Proof

Let $x=(x_{i})$ be lacunary *λ*-statistically convergent to *M*, then, for every $t>0$, $\xi>0$ and $\mu>0$, there exists $i_{0} \in\mathbb{N}$ such that

$$\delta \biggl( \biggl\{ i \in\mathbb{N}: \frac{1}{\lambda_{i}} \biggl\{ j \leq i : \frac{1}{h_{i}} \sum_{j \in I_{i}} M_{j} \biggl( \frac{\nu_{x_{j}-\psi }(t)}{\rho^{(j)}} \biggr) \leq1-\mu \biggr\} \leq1-\xi \biggr\} \biggr)=0, $$

for all $i \geq i_{0}$. Therefore the set

$$B= \biggl\{ i \in\mathbb{N}: \biggl\{ j \leq i: \frac{1}{h_{i}} \sum _{j \in I_{i}} M_{j} \biggl(\frac{\nu_{x_{j}-\psi}(t)}{\rho^{(j)}} \biggr) \leq1-\mu \biggr\} \leq1-\xi \biggr\} \subseteq\{1,2,3,\ldots i_{0}-1 \}. $$

Since *I* is admissible, we have $B \in I$. Hence $I_{\lambda}^{\nu}(\theta )- \lim x=\psi$. □

### Theorem 4.5

*Let* $(X, \nu, \tau)$ *be a PNS*. *If* *x* *is lacunary* *λ*-*statistical convergent*, *then it has a unique limit*.

### Theorem 4.6

*Let* $(X, \nu, \tau)$ *be a PNS*. *If* *x* *is lacunary* *λ*-*statistically convergent*, *then there exists a subsequence* $(x_{m_{k}})$ *of* *x* *such that it is also lacunary* *λ*-*statistically convergent to* *M*.
